# Predictors of betel quid chewing behavior and cessation patterns in Taiwan aborigines

**DOI:** 10.1186/1471-2458-6-271

**Published:** 2006-11-03

**Authors:** Chin-Feng Lin, Jung-Der Wang, Ping-Ho Chen, Shun-Jen Chang, Yi-Hsin Yang, Ying-Chin Ko

**Affiliations:** 1 Institute of Occupational Medicine and Industrial Hygiene, National Taiwan University, College of Public Health and Department of Internal Medicine, National Taiwan University Hospital, Taipei, Taiwan; 2 Department of Health, Executive Yuan, Taiwan; 3 Department of Public Health, Taipei Medical University, Taipei, Taiwan; 4 Division of Environmental Health and Occupational Medicine, National Health Research Institutes, Kaohsiung 807, Taiwan; 5 Department of Public Health, Faculty of Medicine, College of Medicine, Kaohsiung Medical University, Kaohsiung, Taiwan; 6 Graduate Institute of Oral Health Sciences, College of Dental Medicine, Kaohsiung Medical University, Kaohsiung, Taiwan; 7 Statistical Analysis Laboratory, Department of Clinical Research, Kaohsiung Medical University Chung-Ho Memorial Hospital, Taiwan

## Abstract

**Background:**

Betel quid, chewed by about 600 million people worldwide, is one of the most widely used addictive substances. Cessation factors in betel quid chewers are unknown. The present study explores prevalence and the quit rate of betel quid chewing in Taiwan aborigines. Our goal was to delineate potential predictors of chewing cessation.

**Methods:**

A stratified random community-based survey was designed for the entire aborigines communities in Taiwan. A total of 7144 participants were included between June 2003 and May 2004 in this study. Information on sociodemographic characteristics, such as gender, age, obesity, education years, marital status, ethnicity, and habits of betel quid chewing, smoking and drinking was collected by trained interviewers.

**Results:**

The prevalence of betel quid chewers was 46.1%. Betel quid chewing was closely associated with obesity (OR = 1.61; 95% CI: 1.40–1.85). Betel quid chewers were most likely to use alcohol and cigarettes together. Quit rate of betel quid chewers was 7.6%. Betel quid chewers who did not drink alcohol were more likely to quit (OR = 1.89; 95% CI: 1.43–2.50). Alcohol use is a significant factor related to cessation of betel quid chewing, but smoking is not.

**Conclusion:**

Taiwan aborigines have a high prevalence of betel quid chewers and a low quit rate. Alcohol use is strongly association with betel quid chewing. Efforts to reduce habitual alcohol consumption might be of benefit in cessation of betel quid chewing.

## Background

Betel quid consumption is an addictive habit with psychoactive properties, used by an estimated 600 million people worldwide [1-4]. This practice is widespread in Taiwan, with approximately two million habitual users (10% of population) [5]. In 2004, the International Agency for Research on Cancer (IARC) declared chewing of betel quid, by itself, to be a Group 1 carcinogen and the areca nut to be, correspondingly, a Group 1 carcinogen [6]. Chewing betel quid independently contributes to the risk of oropharyngeal cancer [7-9], oral mucosal lesions [10], oral leukoplakia [11], oral submucous fibrosis (OSF) [11], liver cirrhosis [12], hepatocellular carcinoma (HCC) [13], diabetes mellitus [14] and adverse outcomes with use during pregnancy [15,16]. These pertinent health risks highlight the need for cessation of betel quid chewing. Indeed, betel quid chewing is a recognized public health problem in Taiwan; active strategies are necessary to reduce use.

Several factors influence betel quid chewing, including predisposition associated with ethnicity, demographic and psychosocial factors, its accessibility and public policy. Taiwan aborigines have an extensive history of betel quid use. Their habits are dictated by different social and cultural behaviors in both genders. The life expectancy of Taiwan aborigines is one decade lower than the general population [17] and the mortality rate and prevalence of chronic diseases, such as liver cirrhosis, oral cancer and renal failure, are two-to five-folds higher than rest of the Taiwanese population [18]. The estimated prevalence of betel quid chewing in Taiwan aborigines is comparably higher than rest of Taiwanese population (42% for Taiwan aborigines; 6% for Taiwanese population)[5]. Despite studies to investigate the high prevalence of betel quid chewing in Taiwan aborigines, contributing factors and quit patterns between former and current chewers have not been documented. This report provides the first description of prevalence and patterns of cessation in Taiwan aborigines.

## Methods

### Design

A large-scale survey of substance use (betel quid, alcohol and cigarette) among Taiwan aborigines was completed. Data was obtained by interviewing participants in their communities between June 2003 and May 2004.

Approximately 86% of Taiwanese are descended from Han Chinese, whose ancestors migrated 400 years ago from Fukkien and Canton provinces [19]. Taiwan aborigines constitute approximately 2% of the population, rarely mix with Han Chinese and reside in isolated mountainous areas with their own language, customs and social organizations. Genetically, they are markedly different from Han Chinese [20]. There are twelve aborigines tribes in Taiwan and most live in fifty isolated communities. Very few Han Chinese live in aborigines communities unless they work in the community or married aborigines. Due to random sampling, about 9% of participants are Han Chinese. They provide a comparison between aborigines and non-aborigines, because they lived in the same community. A random sampling approach was used with these fifty communities, stratified by gender and age group (>= 18 years). According to our previous study, the prevalence of betel quid chewing is approximately 20%. We randomly selected 150 to 200 persons via a household registry system in each community, obtaining 95% confidence interval (CI) within ± 3% [5]. The prevalence of betel quid chewing is between 1% and 50% in some areas, obtaining the prevalence of 95% CI between ± 1% and 5% [5]. The prevalence of betel quid chewing in Taiwan aborigines males and females is 42.1% and 19.2% respectively, among three tribes [5]. Each participant completed an interview questionnaire with trained nurses, fulfilling our target population of 9124 persons. Questionnaire contents included demographic characteristics, body morphology (height and weight), past and current histories of betel chewing, and cigarette and alcohol use. This study was approved by the Medical Associations for Indigenous People in Taiwan (No. 930010) and agreed by Department of Health, Executive Yuan (No. 0933500309).

### Data analysis

#### Dependent variables

Separate analyses were conducted for betel quid chewing behavior and chewing cessation. The dichotomous dependent variable, betel quid chewing behavior, was coded as betel quid chewers (chewed at least once a week, irrespective of quantity) or never chewers (those who never chewed). The other dependent variable, betel quid chewing cessation, was coded as former chewers (those who quit chewing at least one year prior the survey) or current chewers (continual chewing habit). In addition, betel quid chewers included former chewers and current chewers.

#### Independent variables

Potential explanatory (independent) variables included gender, age, obesity, education, age commenced chewing (years), quids per day, marital status, ethnicity, alcohol consumption, and cigarette smoking. Taiwan aborigines status was classed into three groups, "Taiwan aborigines" when both parents were Taiwan aborigines, "mixed Taiwan aborigines" when one parent was Taiwan aborigines, and "non-Taiwan aborigines" when both parents were not Taiwan aborigines. Body mass index (BMI) was calculated as body weight in kilograms divided by square of body height in meters. Obesity was defined by BMI ≥ 27.8 kg/m^2 ^for men and ≥ 27.3 kg/m^2 ^for women. Continuous variables, including age, education years, age commenced chewing, numbers chewed per day, were divided into two categories, above and below the median cut-off.

### Statistical analyses

Data were entered into a spreadsheet and analyzed using Statistic Analysis Software (SAS release 8.2, Cary, NC, USA). Missing data were excluded. Crude odds ratios (OR) with 95% CI were calculated. To control for potential confounding effects, statistically significant ORs were subsequently examined in the multiple logistic regression model to obtain adjusted OR (aOR). *P *value less than 0.05 or a range of 95% CI that did not include unity, were considered statistically significant.

## Results

A total of 7326 participants completed our questionnaires from aborigines population of 9124. The response rate was 80.3% (7326/9124). All participants were over 18 years of age (mean 45.3 ± 17.0 years). Factors associated with a betel quid chewing habit are shown in Table 1. The prevalence of betel quid chewers (current and former chewers) was 46.1% of total subjects (3291/7144), with the males (53.2%, 2033/3824) higher than females (37.9%, 1258/3320) *p *< 0.05. After adjustment for all factors with a logistic regression model, factors most associated with betel chewing were male, obesity, less education, married status, Taiwan aborigines, mixed-Taiwan aborigines, drinking alcohol and smoking cigarettes (*p *< 0.05). It was noteworthy that the adjusted OR stratified by age showed that older group (>= 45 years) was less likely to have chewing habits.

Table 2 depicts the distribution of demographic characteristics, comparing former chewers to current chewers. The 251 former chewers account for 7.6% in betel quid chewers (251/3291). The quit rate for males is 8.2% (167/2033) and 6.7% (84/1258) for females. In the unadjusted analysis, the crude OR showed those who were older, non-Taiwan aborigines, chewing a smaller amount of quids per day (quids <= 10) and consuming no alcohol to be most likely to quit their chewing habits. Adjusting associations with logistic regression, those older than 45 years and with no alcohol consumption habits had a significantly higher quit rate (*p *< 0.05). However, the Taiwan aborigines were still less likely to quit chewing habits.

Prevalence of current chewers by ethnicity and gender (Table 3), compared to total subjects was 42.6% (3040/7144) and of current betel quid chewers, males and females were 48.8% and 35.4%, respectively. Ethnicity was classified into five groups: both parents are Taiwan aborigines, only father is Taiwan aborigines, only mother is Taiwan aborigines, non-Taiwan aborigines, and others. The highest prevalence of current male chewers was in those whose mother was Taiwan aborigines (55.7%; 64/115). In females, the highest prevalence was among those whose both parents were Taiwan aborigines (39.3%; 1115/2834). The quit rate of betel quid chewing by ethnicity and sex is summarized in Table 4. Excluding others, the highest quit rate (18.5%) was seen in non-Taiwan aborigines males. In contrast, the lowest quit rate was 3.5% among females whose mother is Taiwan aborigines.

Independent effects of alcohol consumption, cigarette smoking and betel quid chewing on cessation were evaluated by stratifying alcohol and cigarette use across the behavior of betel quid chewing (Table 5). For the nondrinker and nonsmoker who chewed betel quid before, the likelihood of cessation increased 1.76-fold over those who drink and smoke. Likelihood was also significantly increased for betel quid chewers who did not drink, but had smoking habits (OR = 2.19; 95% CI: 1.48 – 3.26). After controlling for other factors, similar cessation patterns were observed in the betel quid chewers who had no drinking habits, but did smoke (aOR = 1.99; 95% CI: 1.32 – 2.99).

## Discussion

Research into betel quid chewing and cessation factors is limited. In this large survey, the sociodemographic factors in betel quid chewing behavior and cessation patterns in Taiwan aborigines were examined. Compared to those who never chewed, betel quid chewers were more likely to be male, obese, of lower education, married, Taiwan aborigines or mixed Taiwan aborigines, a drinker and smoker. On the contrary, subjects who were older were less likely to be betel quid chewers.

This study is more reliable than past studies in determining prevalence and betel chewing association factors in Taiwan aborigines, because a randomized and systematic surveillance-style approach was adopted to recruit participants from their own communities.

There were more betel quid chewers among males than females in this study (aOR = 1.14; 95% CI: 1.01–1.30). This might be explained by the higher prevalence of chewing in Taiwanese males [5]. Taiwan aborigines have a higher prevalence of betel quid chewing than Han Chinese [5] and are more likely to be betel quid chewers than non-Taiwan aborigines (aOR = 3.83; 95% CI: 3.01–4.89). In Taiwan, betel quid chewing is widespread; chewers are frequently alcohol drinkers and smokers. After adjusting for other factors, the prevalence of drinking and smoking habits was significantly higher in betel quid chewers than in those who never chewed.

Another important finding is that betel quid chewing is closely associated with obesity. Obesity is a recognized risk factor for chronic diseases, such as diabetes mellitus, cardiovascular diseases, and hypertension. According to previous studies, betel quid chewing can increase body mass index (BMI) or contribute to obesity [21-23] by the betel quid constituents (alkaloid, arecoline and arecaidine) as inhibitors of γ-aminobutyric acid (GABA) receptor [4,24].

The present study is the first estimation of quit rates for betel quid chewing among Taiwan aborigines. Our findings indicate that cessation probability increases with older age group, non-Taiwan aborigines, and those who do not drink. Interestingly, another survey on smoking behavior showed a significantly positive association between smoking cessation and increased age [25]. We observed that former chewers comprise 7.6% of total users. Within this, Taiwan aborigines (7.2%) and mixed Taiwan aborigines (7.0%) had a lower quit rate than non-Taiwan aborigines (17.9%, *p *< 0.05). Apart from the Taiwan aborigines heritage, age group and use of betel quid with alcohol influenced the quit rate. Our study suggested that young Taiwan aborigines or mixed-Taiwan aborigines, who were also alcohol drinkers, found it more difficult to abstain from betel quid chewing. Dr. Wen *et al. *noted the combined effects of cigarette smoking and betel quid chewing in Han Chinese [26] and suggested that betel quid chewing should not to be considered as an isolated issue. Our results have shown the combined effect of betel quid chewing and alcohol consumption also existed in Taiwan aborigines. Therefore, cessation should be viewed conjointly with alcohol consumption.

Clearly, the highest prevalence of both chewing and of quitting chewing was seen in Taiwan aborigines males whose mother was Taiwan aborigines (Table 3 and Table 4). However, in Taiwan aborigines females, this consistent association was not obvious. We suggest that the likelihood of being a current or former chewer was higher in Taiwan aborigines males whose mother is Taiwan aborigines.

Our multivariate analyses noted that alcohol consumption was an independent factor in cessation (Table 5). For the nondrinkers and nonsmokers who chewed betel quid before, their cessation rate was higher than in those who had drinking and smoking habits. Nondrinkers with cigarette smoking had the highest cessation behavior, compared to those who have drinking and smoking habits, even after adjusting for covariates (OR = 1.99; 95% CI = 1.32–2.99). The cessation discrepancy between betel quid chewers with and without alcohol implies that alcohol not consumption have an additional effect on cessation [5]. The main outcome of our study is that efforts to encourage cessation of betel quid chewing will need to include efforts to reduce habitual alcohol consumption. In contrast, reducing cigarette smoking also serves as an important step in reducing betel quid chewing [26].

## Conclusion

Taiwan aborigines have a high prevalence of betel quid chewing and a low quit rate. Betel quid chewing independently contributes to the likelihood of obesity. Alcohol usage is strongly related to betel quid chewing. Efforts to reduce betel quid chewing might benefit from a combined approach targeting both these habits.

## Competing interests

The author(s) declare that they have no competing interests.

## Authors' contributions

CF and JD carried out the studies, participated in the sequence alignment and drafted the manuscript. PH, SJ and YH participated in the design of the study and performed the statistical analysis. YC led this study, and participated in its design and coordination and helped to complete the manuscript. All of the authors participated in the establishment of the system design, and all read and approved the manuscript.

## Pre-publication history

The pre-publication history for this paper can be accessed here:



## Figures and Tables

**Table 1:** Sociodemographic characteristics and betel quid chewing behaviors (N = 7144)

	Betel quid chewers (N = 3291)		Never chewers (N = 3853)		OR^b ^(95% CI)	aOR^c ^(95% CI)
			
	N	(%)^a^	N	(%)^a^		
Gender*						
Male	2033	(53.2)	1791	(46.8)	1.86 (1.69–2.05) †	1.14 (1.01–1.30) †
Female	1258	(37.9)	2062	(62.1)	1.00	1.0
Age (years)*						
>=45	1582	(48.0)	1716	(52.0)	1.15 (1.05–1.27) †	0.84(0.72–0.97) †
<45	1709	(44.4)	2137	(55.6)	1.00	1.0
Obesity*						
Yes	800	(55.8)	633	(44.2)	1.63 (1.45–1.84) †	1.61 (1.40–1.85) †
No	2491	(43.6)	3220	(56.4)	1.00	1.0
Education (years)*						
<=6	1709	(52.8)	1531	(47.3)	1.64 (1.49–1.80) †	2.02 (1.75–2.34) †
>6	1582	(40.5)	2322	(59.5)	1.00	1.0
Marital status*						
Married	2661	(49.1)	2762	(50.9)	1.69 (1.51–1.89) †	1.51 (1.30–1.75) †
Single	602	(36.3)	1057	(63.7)	1.00	1.0
Ethnicity*						
Taiwan aborigines	3018	(49.7)	3059	(50.3)	5.30 (4.28–6.56) †	3.83 (3.01–4.89) †
Mixed-Taiwan aborigines	128	(43.1)	169	(56.9)	4.07 (2.98–5.54) †	3.01 (2.09–4.34) †
Non-Taiwan aborigines	106	(15.7)	569	(84.3)	1.00	1.0
Alcohol consumption*						
Yes	2480	(66.9)	1225	(33.1)	7.05 (6.33–7.84) †	4.50 (4.00–5.07) †
No	731	(22.3)	2545	(77.7)	1.00	1.0
Cigarette smoking*						
Yes	1821	(66.8)	906	(33.2)	4.28 (3.86–4.74) †	3.03 (2.66–3.45) †
No	1363	(32.0)	2899	(68.0)	1.00	1.0

* Significant difference in prevalence of betel quid chewing by Chi-square analysis (*p *< 0.05).

† Significant difference by logistic regression model (*p *< 0.05).

^a ^May not total 100% due to rounding.

^b ^Odds ratios (OR) refer to risk of betel quid chewers (including former chewers and current chewers) versus never chewers according to each variable. OR > 1 indicates a higher likelihood of being a betel quid chewer.

^c ^aOR: adjusted odds ratio for sex, age (years), obesity, education (years), marital status, ethnicity, alcohol consumption, and cigarette smoking by logistic regression model; 95% CI: 95% confidence interval.

**Table 2:** Demographic characteristics in the cessation of betel quid chewing. (N = 3291)

	Betel quid chewers					
	
	Former chewers (N = 251)		Current chewers (N = 3040)		OR^b ^(95% CI)	aOR^c ^(95% CI)
			
	N	(%)^a^	N	(%)^a^		
Gender						
Male	167	(8.2)	1866	(91.8)	1.25(0.95–1.64)	-
Female	84	(6.7)	1174	(93.3)	1.00	
Age (yrs)*						
>=45	157	(9.9)	1425	(90.1)	1.89(1.45–2.47) †	1.84(1.40–2.42) †
<45	94	(5.5)	1615	(94.5)	1.00	1.00
Obesity						
Yes	58	(7.3)	742	(92.8)	0.93(0.69–1.26)	-
No	193	(7.8)	2298	(92.3)	1.00	
Education (years)						
<=6	137	(8.0)	1572	(92.0)	1.12(0.87–1.45)	-
>6	114	(7.2)	1468	(92.8)	1.00	
Age commenced chewing (years)						
>=20	166	(8.0)	1923	(92.1)	1.13(0.87–1.49)	-
<20	85	(7.1)	1117	(92.9)	1.00	
Quids per day*						
<=10	134	(8.8)	1397	(91.3)	1.34(1.04–1.74) †	1.24(0.95–1.62)
>10	117	(6.7)	1643	(93.4)	1.00	1.00
Marital status						
Married	213	(8.0)	2448	(92.0)	1.33(0.93–1.91)	-
Single	37	(6.2)	565	(93.9)	1.00	
Ethnicity*						
Taiwan aborigines	218	(7.2)	2800	(92.8)	0.36(0.21–0.60) †	0.40(0.24–0.68) †
Mixed-Taiwan aborigines	9	(7.0)	119	(93.0)	0.35(0.15–0.80) †	0.47(0.20–1.11)
Non-Taiwan aborigines	19	(17.9)	87	(82.1)	1.00	1.00
Alcohol consumption*						
No	91	(12.5)	640	(87.6)	2.10(1.60–2.76) †	1.89(1.43–2.50) †
Yes	157	(6.3)	2323	(93.7)	1.00	1.00
Cigarette smoking						
No	97	(7.1)	1266	(92.9)	0.89(0.68–1.16)	-
Yes	145	(8.0)	1676	(92.0)	1.00	

* Significant difference in the prevalence of former and current chewers by Chi-square analysis (*p *< 0.05)

† Significant difference by logistic regression model (*P *< 0.05).

^a ^May not total 100% due to rounding.

^b ^Odds ratios (OR) refer to the risk of former chewers versus current chewers according to each variable. OR > 1 indicates a higher likelihood of being a former chewer (quit chewing).

^c ^aOR: adjusted odds ratio for age (years), quids per day, ethnicity and alcohol consumption by logistic regression model; 95% CI: 95% confidence interval.

**Table 3:** Prevalence rate of current betel quid chewers (N = 3040) by ethnicity and by gender

	Prevalence rate of current betel quid chewers (%)	
	
	Males	Females
Ethnicity		
Both parents are Taiwan aborigines	1685/3243 (52.0)	1115/2834 (39.3)
Only father is Taiwan aborigines	22/52 (42.3)	5/36 (13.9)
Only mother is Taiwan aborigines	64/115 (55.7)	28/94 (29.8)
Non-Taiwan aborigines	75/360 (20.8)	12/315 (3.8)
Others	20/54 (37.0)	14/31 (45.2)
		
Subtotal	1866/3824 (48.8)	1174/3320 (35.4)

Total	3040/7144 (42.7)	

**Table 4:** Quit rate of betel quid chewers (N = 3291) by ethnicity and by gender

	Quit rate of betel quid chewers (%)	
	
	Males	Females
Ethnicity		
Both parents are Taiwan aborigines	138/1823 (7.6)	80/1195 (6.7)
Only father is Taiwan aborigines	1/23 (4.4)	1/6 (16.7)
Only mother is Taiwan aborigines	6/70 (8.6)	1/29 (3.5)
Non-Taiwan aborigines	17/92 (18.5)	2/14 (14.3)
Others	5/25 (20)	0/14 (0)
		
Subtotal	167/2033 (8.2)	84/1258 (6.7)

Total	251/3291 (7.6)	

**Table 5:** Effects of alcohol consumption and cigarette smoking on cessation

		Betel quid chewers			
		
		Former chewers (N = 239)	Current chewers (N = 2882)	OR(95%CI)^a^	aOR(95%CI)^b^
Alcohol No					
	Cigarette No	51	390	1.76(1.24–2.50) †	1.25(0.86–1.83)
	Cigarette Yes	38	233	2.19(1.48–3.26) †	1.99(1.32–2.99) †
Alcohol Yes					
	Cigarette No	45	847	0.72(0.50–1.02)	0.59(0.40–1.86) †
	Cigarette Yes	105	1412	1.00	1.00

† Significant difference by logistic regression model (*p *< 0.05).

^a ^Odds ratios (OR) refer to the risk of former chewers versus current chewers according to each variable. OR > 1 indicates a higher likelihood of being a former chewer (quit chewing).

^b ^aOR: adjusted odds ratio for age (years), quids per day, and ethnicity by logistic regression model; 95% CI: 95% confidence interval.
